# Early Appearance of Thyroid Autoimmunity in Children Followed From Birth for Type 1 Diabetes Risk

**DOI:** 10.1210/clinem/dgae478

**Published:** 2024-07-12

**Authors:** Berglind Jonsdottir, Joanna L Clasen, Kendra Vehik, Åke Lernmark, Markus Lundgren, Ezio Bonifacio, Desmond Schatz, Anette-Gabriele Ziegler, William Hagopian, Marian Rewers, Richard McIndoe, Jorma Toppari, Jeffrey Krischer, Beena Akolkar, Andrea Steck, Riitta Veijola, Michael J Haller, Helena Elding Larsson, the TEDDY Study Group, Marian Rewers, Kimberly Bautista, Judith Baxter, Daniel Felipe-Morales, Brigitte I Frohnert, Marisa Stahl, Isabel Flores Garcia, Patricia Gesualdo, Sierra Hays, Michelle Hoffman, Randi Johnson, Rachel Karban, Edwin Liu, Leila Loaiza, Jill Norris, Holly O’Donnell, Loana Thorndahl, Andrea Steck, Kathleen Waugh, Jorma Toppari, Olli G Simell, Annika Adamsson, Suvi Ahonen, Mari Åkerlund, Sirpa Anttila, Leena Hakola, Anne Hekkala, Tiia Honkanen, Teija Hurskainen, Heikki Hyöty, Jorma Ilonen, Saori Itoshima, Minna Jokipolvi, Sanna Jokipuu, Taru Karjalainen, Leena Karlsson, Jukka Kero, Marika Korpela, Jaakko J Koskenniemi, Miia Kähönen, Mikael Knip, Minna-Liisa Koivikko, Katja Kokkonen, Merja Koskinen, Mirva Koreasalo, Kalle Kurppa, Salla Kuusela, Jarita Kytölä, Mia Laakso, Jutta Laiho, Tiina Latva-aho, Siiri Leisku, Laura Leppänen, Katri Lindfors, Maria Lönnrot, Elina Mäntymäki, Markus Mattila, Maija Miettinen, Tiina Niininen, Sari Niinistö, Noora Nurminen, Sami Oikarinen, Hanna-Leena Oinas, Paula Ollikainen, Zhian Othmani, Sirpa Pohjola, Solja Raja-Hanhela, Jenna Rautanen, Anne Riikonen, Minna Romo, Juulia Rönkä, Nelli Rönkä, Satu Simell, Aino Tihinen, Päivi Tossavainen, Mari Vähä-Mäkilä, Eeva Varjonen, Riitta Veijola, Irene Viinikangas, Silja Vilmi, Suvi M Virtanen, Richard McIndoe, Desmond Schatz, Diane Hopkins, Michael Haller, Melissa Gardiner, Ashok Sharma, Laura Jacobsen, Percy Gordon, Jennifer Hosford, Sharon Maina, Chelsea Salmon, Anette G Ziegler, Ezio Bonifacio, Cigdem Gezginci, Willi Grätz, Anja Heublein, Sandra Hummel, Annette Knopff, Sibylle Koletzko, Claudia Ramminger, Roswith Roth, Jennifer Schmidt, Marlon Scholz, Joanna Stock, Katharina Warncke, Lorena Wendel, Christiane Winkler, Helmholtz Zentrum München, Forschergruppe Diabetes, Klinikum rechts der Isar, Åke Lernmark, Daniel Agardh, Carin Andrén Aronsson, Rasmus Bennet, Corrado Cilio, Susanne Dahlberg, Malin Goldman Tsubarah, Emelie Ericson-Hallström, Lina Fransson, Emina Halilovic, Susanne Hyberg, Berglind Jonsdottir, Naghmeh Karimi, Helena Elding Larsson, Marielle Lindström, Markus Lundgren, Marlena Maziarz, Jessica Melin, Kobra Rahmati, Anita Ramelius, Falastin Salami, Anette Sjöberg, Evelyn Tekum Amboh, Carina Törn, Ulrika Ulvenhag, Terese Wiktorsson, Åsa Wimar, William A Hagopian, Michael Killian, Claire Cowen Crouch, Jennifer Skidmore, Trevor Bender, Megan Llewellyn, Cody McCall, Arlene Meyer, Jocelyn Meyer, Denise Mulenga, Nole Powell, Jared Radtke, Shreya Roy, Preston Tucker, Dorothy Becker, Margaret Franciscus, MaryEllen Dalmagro-Elias Smith, Ashi Daftary, Mary Beth Klein, Chrystal Yates, Jeffrey P Krischer, Rajesh Adusumali, Sarah Austin-Gonzalez, Maryouri Avendano, Sandra Baethke, Brant Burkhardt, Martha Butterworth, Nicholas Cadigan, Joanna Clasen, Kevin Counts, Laura Gandolfo, Jennifer Garmeson, Veena Gowda, Christina Karges, Shu Liu, Xiang Liu, Kristian Lynch, Jamie Malloy, Lazarus Mramba, Cristina McCarthy, Jose Moreno, Hemang M Parikh, Cassandra Remedios, Chris Shaffer, Susan Smith, Noah Sulman, Roy Tamura, Dena Tewey, Henri Thuma, Michael Toth, Ulla Uusitalo, Kendra Vehik, Ponni Vijayakandipan, Melissa Wroble, Jimin Yang, Kenneth Young, Michael Abbondondolo, Lori Ballard, Rasheedah Brown, David Cuthbertson, Stephen Dankyi, Christopher Eberhard, Steven Fiske, David Hadley, Kathleen Heyman, Belinda Hsiao, Francisco Perez Laras, Hye-Seung Lee, Qian Li, Laura Smith, William Hagopian, Jared Radtke, Preston Tucker, Clive H Wasserfall, William E Winter, David L Pittman, Chris Deigan, Beena Akolkar, Thomas Briese, Todd Brusko, Teresa Buckner, Suzanne Bennett Johnson, Eoin McKinney, Tomi Pastinen, Steffen Ullitz Thorsen, Eric Triplett

**Affiliations:** Department of Pediatrics, The Children's Hospital Iceland, 101 Reykjavik, Iceland; Department of Clinical Science Malmö, Lund University, 20502 Malmö, Sweden; Health Informatics Institute, Morsani College of Medicine, University of South Florida, Tampa, FL 33612, USA; Health Informatics Institute, Morsani College of Medicine, University of South Florida, Tampa, FL 33612, USA; Department of Clinical Science Malmö, Lund University, 20502 Malmö, Sweden; Department of Clinical Science Malmö, Lund University, 20502 Malmö, Sweden; Center for Regenerative Therapies Dresden, Faculty of Medicine, Technische Universität Dresden, 01307 Dresden, Germany; Department of Pediatrics, University of Florida, Gainesville, FL 32610, USA; German Center for Environmental Health, Institute of Diabetes Research, Helmholtz Munich, 80939 Munich, Germany; Forschergruppe Diabetes, School of Medicine, Klinikum rechts der Isar, Technical University Munich, 81675 Munich, Germany; Forschergruppe Diabetes e.V. at Helmholtz Munich, German Research Center for Environmental Health, 80939 Munich, Germany; Pacific Northwest Research Institute, Seattle, WA 98122, USA; Barbara Davis Center for Childhood Diabetes, University of Colorado, Aurora, CO 80045, USA; Center for Biotechnology and Genomic Medicine, Medical College of Georgia, Augusta University, Augusta, GA 30912, USA; Department of Pediatrics, Turku University Hospital, 20520 Turku, Finland; Institute of Biomedicine, Research Centre for Integrative Physiology and Pharmacology and Centre for Population Health Research, University of Turku, 20520 Turku, Finland; Health Informatics Institute, Morsani College of Medicine, University of South Florida, Tampa, FL 33612, USA; Division of Diabetes, Endocrinology, & Metabolic Diseases, National Institutes of Diabetes and Digestive and Kidney Diseases, Bethesda, MD 20892, USA; Barbara Davis Center for Childhood Diabetes, University of Colorado, Aurora, CO 80045, USA; Department of Pediatrics, Research Unit of Clinical Medicine, Medical Research Center Oulu, Oulu University Hospital and University of Oulu, FI-90014 Oulu, Finland; Department of Pediatrics, University of Florida, Gainesville, FL 32610, USA; Department of Clinical Science Malmö, Lund University, 20502 Malmö, Sweden; Department of Pediatrics, Skåne University Hospital, 20502 Malmö, Sweden

**Keywords:** thyroid autoimmunity, children, autoimmune thyroid disease

## Abstract

**Context:**

Autoantibodies to thyroid peroxidase (TPOAb) and thyroglobulin (TgAb) define preclinical autoimmune thyroid disease (AITD), which can progress to either clinical hypothyroidism or hyperthyroidism.

**Objective:**

We determined the age at seroconversion in children genetically at risk for type 1 diabetes.

**Methods:**

TPOAb and TgAb seropositivity were determined in 5066 healthy children with human leukocyte antigen (HLA) DR3- or DR4-containing haplogenotypes from The Environmental Determinants of Diabetes in the Young (TEDDY) study. Children seropositive on the cross-sectional initial screen at age 8 to 13 years had longitudinally collected samples (from age 3.5 months) screened retrospectively and prospectively for thyroid autoantibodies to identify age at seroconversion. The first-appearing autoantibody was related to sex, HLA genotype, family history of AITD, and subsequent thyroid dysfunction and disease.

**Results:**

The youngest appearance of TPOAb and TgAb was age 10 and 15 months, respectively. Girls had higher incidence rates of both autoantibodies. Family history of AITD was associated with a higher risk of TPOAb hazard ratio (HR) 1.90; 95% CI, 1.17-3.08; and TgAb HR 2.55; 95% CI, 1.91-3.41. The risk of progressing to hypothyroidism or hyperthyroidism was not different between TgAb and TPOAb, but children with both autoantibodies appearing at the same visit had a higher risk compared to TPOAb appearing first (HR 6.34; 95% CI, 2.72-14.76).

**Conclusion:**

Thyroid autoantibodies may appear during the first years of life, especially in girls, and in children with a family history of AITD. Simultaneous appearance of both autoantibodies increases the risk for hypothyroidism or hyperthyroidism.

Autoantibodies to thyroid peroxidase (TPOAb) and thyroglobulin (TgAb) define preclinical autoimmune thyroid disease (AITD), a common endocrine disease characterized by T- and B-lymphocyte infiltration of the thyroid gland. Over time, many patients with AITD progress to develop hypothyroidism or hyperthyroidism, which we define as clinical thyroid disease (1, 2). Either autoantibody may be present independently of the other, not all patients with thyroid disease are autoantibody positive, and not all autoantibody positive individuals develop hypothyroidism or hyperthyroidism (3, 4). The immune-mediated destruction of the thyroid is likely initiated by environmental factors in individuals who are genetically susceptible to autoimmunity (5). AITD is thus more common in patients with other autoimmune diseases or with a family history of autoimmune disease (6). Furthermore, AITD is diagnosed 5 to 10 times more often in women compared to men, with a slightly lower female-to-male ratio, 4:1, in children and adolescents (7). Although hypothyroidism is the most common clinical presentation of individuals with AITD, hyperthyroidism can occur in patients with TPOAb and TgAb. Therefore, we include both hypothyroidism and hyperthyroidism in our definition of individuals with clinical thyroid disease.

The Environmental Determinants of Diabetes in the Young (TEDDY) study follows children from the general population at increased genetic risk for type 1 diabetes, tested at birth or in the neonatal period for human leukocyte antigen (HLA) genotypes (8). The co-occurrence of type 1 diabetes and AITD is well documented, but less is known about the timing of the first appearance of thyroid autoantibodies in healthy children with increased genetic risk for type 1 diabetes. The aims of this study were to examine the TEDDY population for (1) the incidence of TPOAb and TgAb by age, (2) associations of key demographic factors with risk of thyroid autoimmunity, (3) the co-occurrence of TPOAb and TgAb and progression from single to double autoantibody positivity, and (4) the relation of autoimmunity to abnormal thyrotropin (TSH) and risk of clinical thyroid disease. The primary outcome in this study was thyroid autoimmunity, while the 2 secondary outcomes were abnormal TSH and clinically diagnosed thyroid disease, defined by documentation of hypo/hyperthyroidism by International Classification of Diseases, Tenth Revision (ICD-10) codes and/or use of thyroid medication.

## Materials and Methods

### Study Design

TEDDY is a multinational prospective study with 3 centers in the United States (Washington State, Colorado, and Georgia) and 3 centers in Europe (Finland, Germany, and Sweden). The aim is to identify environmental factors that initiate or protect against not only islet autoimmunity and type 1 diabetes, but also concomitant celiac (9) and thyroid autoimmunity and disease in children at increased genetic risk for type 1 diabetes based on their HLA-DR-DQ genotype (8, 10). Enrolled children were followed from birth to age 15 years or until type 1 diabetes diagnosis. Clinical visits and serum sample collection occurred quarterly until age 4 years, and then every 6 months until age 15 years. Children positive for islet autoantibodies continued follow-up every 3 months regardless of age (11). Samples were collected locally, kept at −80 °C in aliquots, and sent every second week on dry ice to the TEDDY Repository managed by Fisher Biosciences (12). An aliquot was sent on dry ice from the TEDDY Repository directly to the Gainesville, Florida, laboratory without thawing the samples.

**Figure 1.**
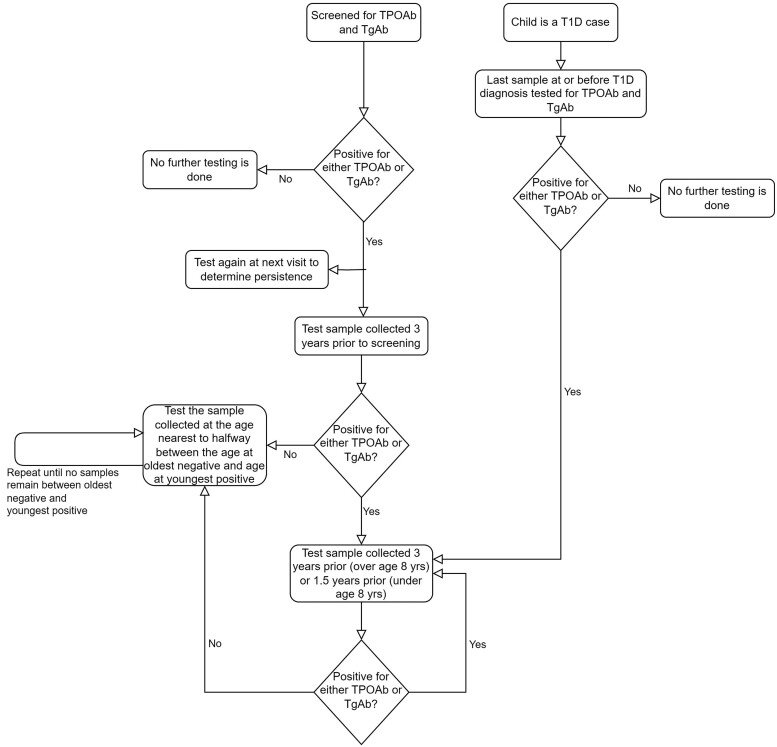
The algorithm for sample selection for testing of autoantibodies to thyroid peroxidase (TPOAb) and thyroglobulin (TgAb) in the TEDDY study. Children actively enrolled and aged 8 years or older in 2016 were screened at the next scheduled clinical visit, while younger children were screened when they reached age 8 years. All children diagnosed with type 1 diabetes were eligible for TPOAb and TgAb testing in the last sample collected at or before diagnosis.

In 2004 to 2010, TEDDY screened 424 788 newborns, and enrolled 8676 children (8). The TEDDY study was approved by local institutional review boards or European ethics committees. Written informed consent was obtained for all study participants from a parent or primary caretaker, separately, for genetic screening and enrollment to participate in the study. The primary outcome in the present analysis was thyroid autoimmunity, defined by the presence of TPOAb or TgAb. Beginning in March 2016, children were initially screened for TPOAb and TgAb at age 8 years or older (up to 13 years). Younger, actively enrolled children were tested when they reached age 8, which occurred as late as 2018, making up a total of 5066 children. This visit when thyroid autoantibody screening was first conducted is hereafter referred to as the screening visit. The screening results were used as a base to perform further testing of samples and construct a retrospective analytical cohort at enrollment (age 3 months). Children who were negative for both autoantibodies at the screening visit were assumed to be negative at all previous time points back to study enrollment. If a child tested positive for either TPOAb or TgAb at the screening visit, both autoantibodies were measured again at the next scheduled visit to confirm the positive test, and a recursive algorithm was applied to retrospective samples to identify the earliest age of thyroid autoantibody positivity (Fig. 1).

Children who were diagnosed with type 1 diabetes before thyroid autoantibody screening commenced (n = 426) were similarly tested retrospectively starting at the last sample prior to type 1 diabetes diagnosis.

Secondary outcomes were (1) thyroid dysfunction, defined as abnormal TSH based on measurements of screening samples positive for TPOAb or TgAb, and (2) clinical thyroid disease. Diagnosis of clinical thyroid disease was carried out outside the purview of the TEDDY study; however, per the study protocol, diagnoses were recorded in a diary book and subsequently reported to TEDDY study staff at the next clinic visit. Study nurses translated reported diagnoses according to the ICD-10. Families similarly reported all medications used by the child. In the present analysis, hypothyroidism cases included children with a reported diagnosis of hypothyroidism (ICD-10 code E03.9 or E06.3) or use of hypothyroidism medication (thyroxine or triiodothyronine), excluding children with congenital hypothyroidism. Likewise, hyperthyroidism cases included children with a reported diagnosis of hyperthyroidism (ICD-10 codes E05.0, E05.8, or E05.9) or use of hyperthyroidism medication (methimazole, carbimazole, dipyrone, radioactive iodine, or propylthiouracil). Clinical thyroid disease cases included both hypothyroidism and hyperthyroidism.

### Measurements of Thyroid Autoantibodies and Thyrotropin

Serum TPOAb (Kronus catalog No. KR6210, RRID:AB_3095086) and TgAb (Kronus catalog No. KR6270, RRID:AB_3095085) were measured by radioimmunoassay at the Pathology Laboratories, University of Florida, US. Final quantitation was calculated using a 5-point cubic spline, log/linear fit algorithm. Values greater than 1.0 U/mL were reported as positive. The cutoff was established by the kit vendor (Kronus). Most screening-visit samples were tested within 30 days of collection, while retrospective samples were retrieved from long-term storage. Prior studies have shown TPOAb and TgAb to be stable for upward of 12 years in storage (13, 14).

TSH was measured at the same laboratory using a Siemens Immulite 2000Xpi analyzer, a solid-phase, 2-sited chemiluminescent immunometric assay (Siemens catalog No. L2KTS2, RRID:AB_3095056). Values greater than 4.0 μIU/mL were considered abnormally high, and values less than 0.4 μIU/mL were considered abnormally low. Coefficient of variation summaries for the TPOAb, TgAb, and TSH assays are shown in Table 1.

**Table 1.** Coefficients of variation at representative mean levels for autoantibodies to thyroid peroxidase, thyroglobulin, and thyrotropin assays

Analyte	Mean, µIU/mL	CV%
TSH	0.42	7.1
TSH	4.89	5.5
TSH	31.64	8
**Analyte**	**Mean, U/mL**	**CV%**
TgAb	0.73	15.1
TgAb	2.52	6.3
TgAb	5.34	7.1
TPOAb	0.66	6.1
TPOAb	3.35	5.1
TPOAb	8.39	7.7

Abbreviations: CV, coefficient of variation; TgAb, autoantibodies to thyroglobulin; TPOAb, autoantibodies to thyroid peroxidase; TSH, thyrotropin.

### Statistical Analysis

Primary analyses included all children tested at the screening visit. Persistent positive status was defined as being positive for the same autoantibody at 2 consecutive visits, and the date of persistent positivity was the draw date of the first of the 2 positives. Children positive for TPOAb or TgAb prior to the visit at age 12 months who did not have a negative result before the first positive were investigated for potential transient positivity due to maternal autoantibodies. If maternal AITD was reported in the family history questionnaire, TPOAb and TgAb results were considered missing at visits prior to the first negative or 12 months, whichever was earlier. Defined by which autoantibody was present when the child first became persistent positive, children were classified as TPOAb-first, TgAb-first, both-first, or thyroid autoantibody negative.

Cox proportional hazards regression models assessed associations between autoantibodies and sex, HLA genotype, and family history. The baseline hazard was stratified by country in all models, and the proportional hazards assumption was assessed with Schoenfeld residuals. Children were followed from birth to first thyroid autoantibody appearance or the screening visit. Sensitivity analyses accounting for the interval-censored data-generating process were compared to the Cox regression models. Modified Poisson regression (15) was used to examine factors associated with risk of progressing from 1 to 2 thyroid autoantibodies. Associations of TSH with first-appearing autoantibody and with autoantibody positivity at the screening visit were also assessed with modified Poisson regression models, excluding children with AITD diagnosed prior to the screening visit. Hazard ratios (HRs) were calculated to assess the association of first-appearing autoantibody with risk of progressing from AITD to clinical thyroid disease, and a sensitivity analysis was conducted in which clinical thyroid disease was defined only by reported medication use and not by ICD-10 codes. Age-specific and cumulative incidence were determined in the primary cohort as well as among children who were tested retrospectively at or before type 1 diabetes diagnosis. Person-years for age-specific incidence was determined based on the number of children (risk-set) with a sample available within the specified age range.

A *P* value of less than .05 was considered to indicate statistical significance, without adjustment for multiple testing. Data wrangling was performed in SAS version 9.4. Analyses were performed with R version 4.3.2 using packages survival (16) version 3.5-7, icenReg (17) version 2.0.15, and survminer version 0.4.9.

## Results

Through October 31, 2022, of 5066 children screened for thyroid autoantibodies at age 8 to 13 years (2492 girls and 2574 boys; Table 2), 385 (7.6%) were positive for either TPOAb or TgAb, with a median age at first appearance of 6.1 years. Sequential retrospective analysis of these children demonstrated that the earliest appearance of TPOAb-first was at age 10 months, with cumulative incidence of 0.3% at 2 years, and 1.0% at 6 years. Among those with TgAb first, the earliest appearance was at age 15 months, with cumulative incidence of 0.2% at 2 and 2.2% at 6 years. Already at age 2 years, 23 (0.5%) children were positive for either autoantibody.

**Table 2.** Characteristics of children tested for thyroid autoantibodies in the TEDDY study

	Tested at screening visit			At or before type 1 diabetes diagnosis		
	Overall	Boys	Girls	Overall	Boys	Girls
n	5066	2574	2492	424	233	191
Follow-up time, median (IQR), y	9.0 (8.1-10.2)	9.0 (8.1-10.3)	9.0 (8.1-10.2)	5.9 (2.2-9.8)	6.3 (2.4-9.9)	5.1 (2.1-9.3)
TPOAb+ or TgAb+, N (%)	385 (7.6)	118 (4.6)	267 (10.7)	41 (9.7)	15 (6.4)	26 (13.6)
TPOAb+ or TgAb + youngest age at first appearance, median (IQR), y	6.1 (3.7-8.0)	6.0 (3.4-8.7)	6.1 (3.8-7.9)	4.7 (3.0-7.2)	6.0 (3.6-9.0)	4.3 (3.0-6.5)
TPOAb-only first, N (%)	102 (2.0)	30 (1.2)	72 (2.9)	8 (1.9)	3 (1.3)	5 (2.6)
TgAb-only first, N (%)	251 (5.0)	82 (3.2)	169 (6.8)	25 (5.9)	10 (4.3)	15 (7.9)
TPOAb and TgAb first, N (%)	32 (0.6)	6 (0.2)	26 (1.0)	8 (1.9)	2 (0.9)	6 (3.1)
Country, N (%)						
USA	1961 (38.7)	1004 (39.0)	957 (38.4)	161 (38.0)	87 (37.3)	74 (38.7)
Finland	1161 (22.9)	577 (22.4)	584 (23.4)	109 (25.7)	60 (25.8)	49 (25.7)
Germany	275 (5.4)	152 (5.9)	123 (4.9)	41 (9.7)	20 (8.6)	21 (11.0)
Sweden	1669 (32.9)	841 (32.7)	828 (33.2)	113 (26.7)	66 (28.3)	47 (24.6)
HLA, N (%)						
Heterozygous	2983 (58.9)	1507 (58.5)	1476 (59.2)	305 (71.9)	170 (73.0)	135 (70.7)
DR4/4	993 (19.6)	482 (18.7)	511 (20.5)	79 (18.6)	41 (17.6)	38 (19.9)
DR3/3	1090 (21.5)	585 (22.7)	505 (20.3)	40 (9.4)	22 (9.4)	18 (9.4)
Family history of autoimmune thyroid disease, N (%)*^a^*	616 (12.3)	314 (12.3)	302 (12.2)	50 (12.4)	26 (11.6)	24 (13.4)
Clinical thyroid disease	88 (1.7)	30 (1.2)	58 (2.3)			

Abbreviations: HLA, human leukocyte antigen; IQR, interquartile range; TgAb, autoantibodies to thyroglobulin; TPOAb, autoantibodies to thyroid peroxidase; TSH, thyrotropin.

^*a*^n = 41 Children at the initial visit and n = 21 children at or before type 1 diabetes were missing family history of autoimmune thyroid disease.

**Figure 2.**
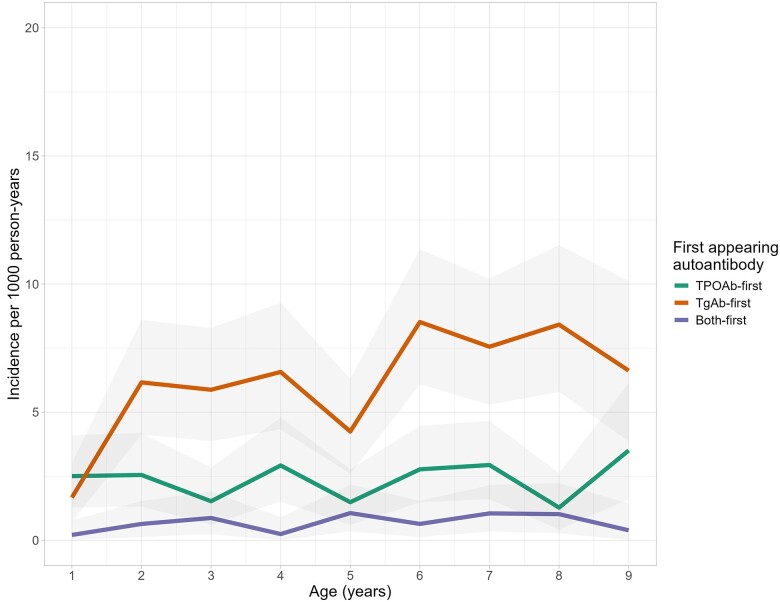
Age-specific incidence rates and 95% CIs for autoantibodies to thyroid peroxidase (TPOAb)-first (green), thyroglobulin (TgAb)-first (orange), and both-first (purple) in 1-year intervals among 5066 children in the TEDDY study. The age 1 interval includes 1.0 years to younger than 2 years, and likewise for subsequent intervals. Incidence rates (per 1000 person-years) of TPOAb-first and TgAb-first at age 2 were 2.5 (95% CI, 1.3-4.2) and 6.2 (95% CI, 4.1-8.6), respectively, and at age 6, 2.8 (95% CI, 1.5-4.5), and 8.5 (95% CI. 6.1-11.4), respectively. Ages 10 to 13 years are not shown because estimates are unstable due to small sample sizes at those ages.

Incidence rates (per 1000 person-years) of TPOAb-first and TgAb-first at age 2 years were 2.5 and 6.2, respectively; and at age 6 years, 2.8 and 8.5, respectively (Fig. 2).

### Overlap and Order of Appearance of Thyroid Autoantibodies

Among 102 children who developed TPOAb first, 54 (53%) later tested persistently positive for TgAb. Similarly, among 251 children with TgAb-first, 120 (48%) later tested persistently positive for TPOAb. In 353 children initially persistently positive for only one autoantibody, there was no indication that progression to persistent positivity for the second autoantibody was associated with sex, HLA genotype, family history, or type of first-appearing autoantibody.

### Risk Factors for Thyroid Autoimmunity

**Figure 3.**
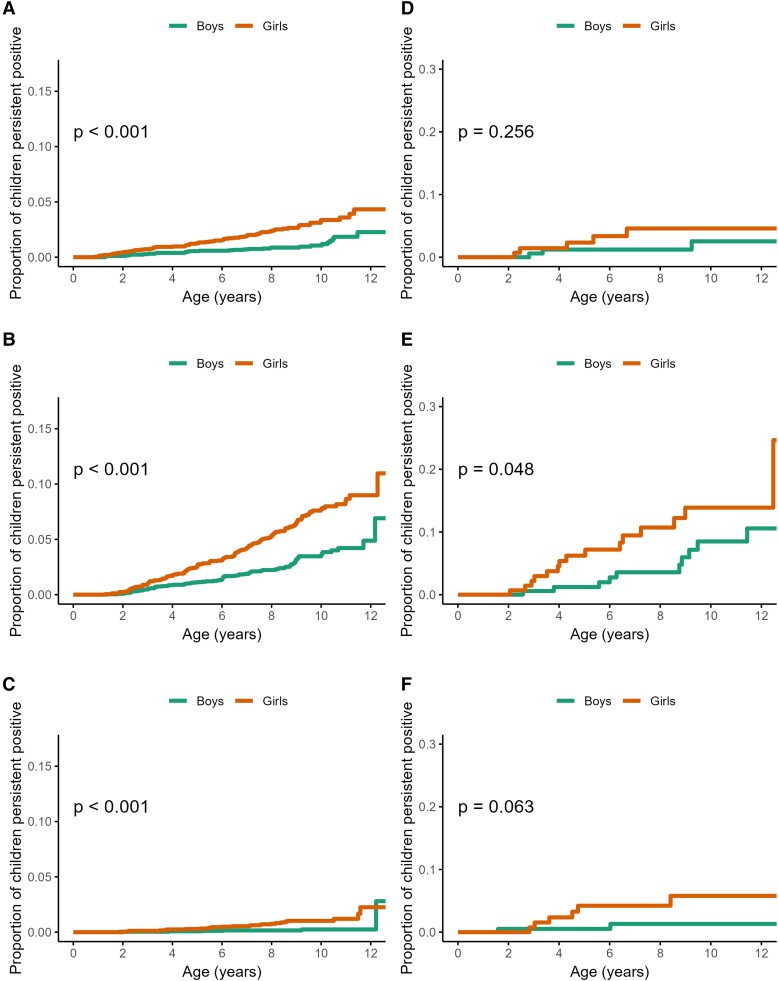
Sex-stratified cumulative incidence and log-rank test *P* value among 5066 children in the TEDDY study of A, autoantibodies to thyroid peroxidase (TPOAb)-first; B, thyroglobulin (TgAb)-first; C, both-first, and among 424 children at or before type 1 diabetes diagnosis of D TPOAb-first; E, TgAb-first; and F, both first.

**Figure 4.**
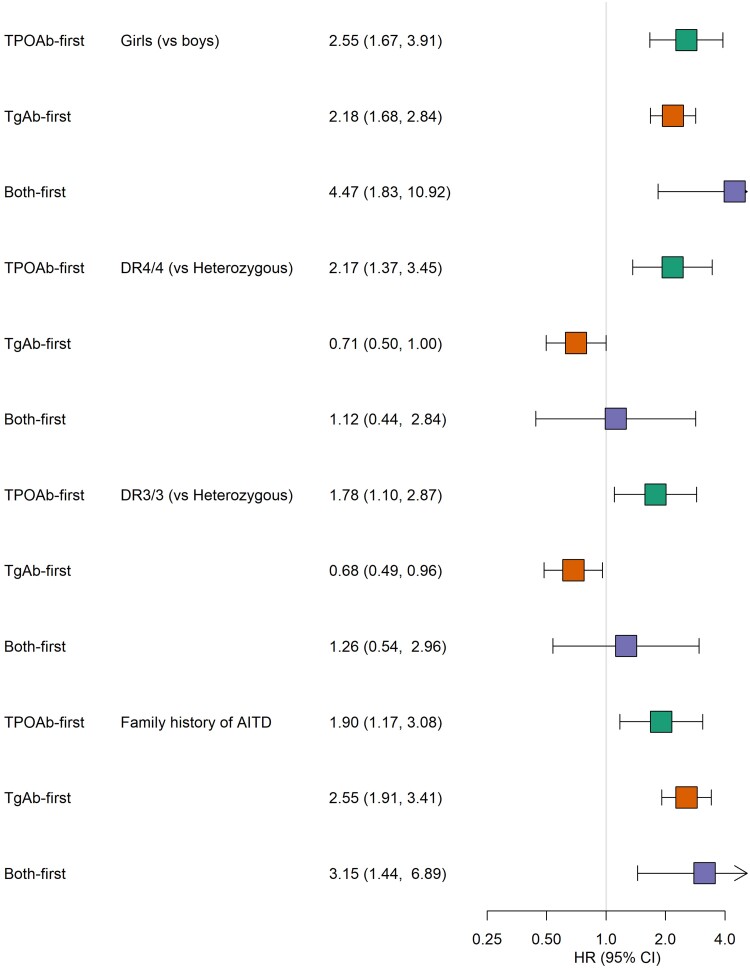
Forest plot of hazard ratios and 95% CIs for associations of key covariates with risk of autoantibodies to thyroid peroxidase (TPOAb)-first (green); thyroglobulin (TgAb)-first (orange), and both first (purple) among 5025 children in the TEDDY study.

Incidence was higher among girls (Fig. 3, panels A-C; *P* < .001 for TPOAb first and TgAb first), a trend that was seen even at young ages: the age-specific incidence rate (per 1000 person-years) of TgAb first at 3 years was 7.7 (95% CI, 4.5-11.7) among girls, and 4.2 (95% CI, 2.0-7.2) among boys. Risks of TPOAb first (HR 2.55; 95% CI, 1.67-3.91; *P* < .001) and TgAb first (HR 2.18; 95% CI, 1.68-2.84; *P* < .001) were more than 2-fold higher for girls compared to boys (Fig. 4).

Risk of TPOAb first was higher among both DR4/4 and DR3/3 homozygous children compared to those with a heterozygous HLA genotype (HR 2.17; 95% CI, 1.37-3.45; *P* < .001, and HR 1.78; 95% CI, 1.10-2.87; *P* = .018, respectively). In contrast, risk of TgAb first was lower for DR4/4 (HR 0.71; 95% CI, 0.50-1.00; *P* = .0497) and DR3/3 (HR 0.68; 95% CI, 0.49-0.96; *P* = .030) compared to HLA heterozygous children. A total of 12% of the children had a first-degree relative with AITD (see Table 2), while the frequency of family history was more than 20% among persistently positive children (Table 3). Family history was associated with a higher risk both of TPOAb first (HR 1.90; 95% CI, 1.17-3.08; *P* = .010) and TgAb first (HR 2.55; 95% CI, 1.91-3.41; *P* < .001; see Fig. 4). Furthermore, the risks of TPOAb first (HR 3.78; 95% CI, 1.65-8.66; *P* = .002) and TgAb first (HR 3.34; 95% CI, 1.94-5.76; *P* < .001) were notably higher if the father had AITD, compared to fathers without AITD. The risk if the mother had AITD was less pronounced (TPOAb-first HR 1.74; 95% CI, 1.03-2.93; *P* = .04; TgAb-first HR 2.19; 95% CI, 1.60-3.01; *P* < .001).

**Table 3.** Demographic factors stratified by first-appearing autoantibody

		TPOAb first	TgAb first	Both first	Thyroid autoantibody negative
n		102	251	32	4681
Sex, N (%)	Male	30 (29.4)	82 (32.7)	6 (18.8)	2456 (52.5)
	Female	72 (70.6)	169 (67.3)	26 (81.2)	2225 (47.5)
HLA, N (%)	DR3/3	28 (27.5)	42 (16.7)	8 (25.0)	1012 (21.6)
	DR4/4	31 (30.4)	39 (15.5)	6 (18.8)	917 (19.6)
	DR3/4	31 (30.4)	124 (49.4)	9 (28.1)	1783 (38.1)
	DR4/8	10 (9.8)	38 (15.1)	7 (21.9)	806 (17.2)
	FDR specific	2 (2.0)	8 (3.2)	2 (6.2)	163 (3.5)
Family history of autoimmune thyroid disease	No	81 (79.4)	189 (75.3)	23 (71.9)	4116 (88.7)
	Yes	21 (20.6)	62 (24.7)	9 (28.1)	524 (11.3)
Country, N (%)	USA	49 (48.0)	104 (41.4)	15 (46.9)	1793 (38.3)
	Finland	20 (19.6)	69 (27.5)	9 (28.1)	1063 (22.7)
	Germany	5 (4.9)	5 (2.0)	1 (3.1)	264 (5.6)
	Sweden	28 (27.5)	73 (29.1)	7 (21.9)	1561 (33.3)

Abbreviations: HLA, human leukocyte antigen; FDR, first-degree relative; TgAb, autoantibodies to thyroglobulin; TPOAb, autoantibodies to thyroid peroxidase; TSH, thyrotropin.

**Figure 5.**
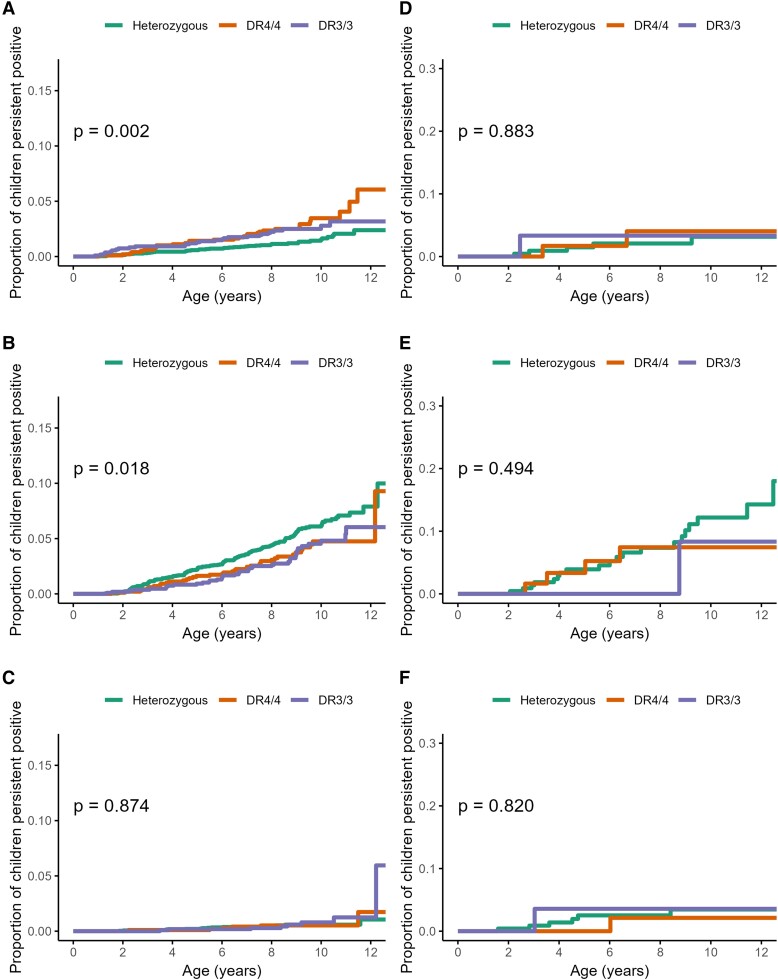
Cumulative incidence and log-rank test *P* value among 5066 children in the TEDDY study of A, autoantibodies to thyroid peroxidase (TPOA)-first; B, thyroglobulin (TgAb)-first; and C, both first; and among 424 children at or before type 1 diabetes diagnosis of D, TPOA-first; and E, TgAb-first; and F, both first. Stratified by human leukocyte antigen genotype.

**Figure 6.**
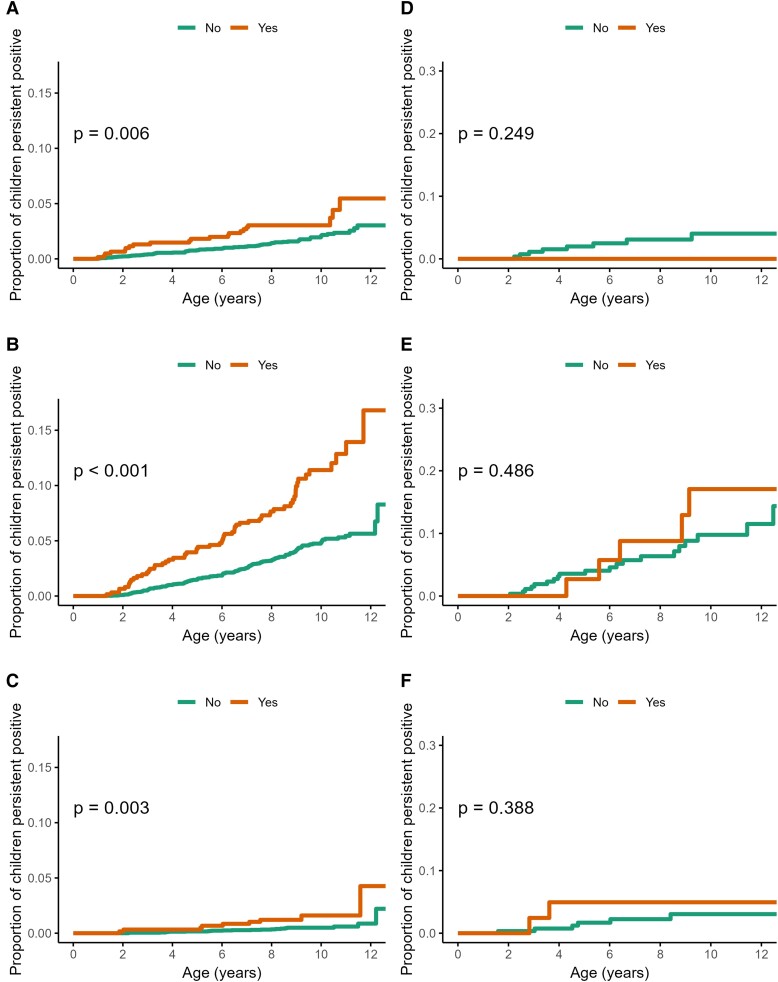
Cumulative incidence and log-rank test *P* value among 5025 children in the TEDDY study of A, autoantibodies to thyroid peroxidase (TPOA)-first; B, thyroglobulin (TgAb)-first; and C, both first; and among 403 children at or before type 1 diabetes diagnosis of D, TPOA-first; E, TgAb-first; and F, both first. Stratified by family history of autoimmune thyroid disease.

Trends in cumulative incidence by first-appearing autoantibody are shown in Fig. 3 and Figs. 5 and 6 (panels A-C). Across all analyzed potential risk factors, there were no notable differences in the estimates or their precision from models accounting for the interval-censored data-generating process.

### Thyrotropin and Clinical Thyroid Disease

Among 371 persistently positive children without a clinical thyroid disease diagnosis prior to the screening visit, 46 had thyroid dysfunction; 43 had high TSH (>4.0 μIU/mL), and 3 had low TSH (<0.4 μIU/mL). Among children positive both for TPOAb and TgAb at the screening visit, 17% had high TSH and 1% had low TSH (Table 4). We did not find evidence of a difference in the risk of high TSH between TPOAb-only and TgAb-only positivity at the screening visit (relative risk [RR] 0.95; 95% CI, 0.28-3.24; *P* = .933), while both-positive compared to TPOAb-only increased the risk of high TSH (RR 3.29; 95% CI, 1.17-9.20; *P* = .023). There was no evidence of an association between first-appearing autoantibody and risk of high TSH.

**Table 4.** Thyrotropin (TSH) results by first-appearing thyroid autoantibody and by thyroid autoantibodies at the time of TSH measurement among 371 children positive for at least one thyroid autoantibody and without an existing clinical thyroid disease diagnosis in the TEDDY study

		High TSH	Low TSH	Normal TSH
	Total	43 (12%)	3 (1%)	325 (88%)
First-appearing autoantibody	TPOAb first	12 (12%)	2 (2%)	83 (86%)
	TgAb first	26 (11%)	1 (0%)	219 (89%)
	Both first	5 (18%)	0 (0%)	23 (82%)
Autoantibodies at time of TSH draw	Only TPOAb positive	3 (7%)	0 (0%)	43 (93%)
	Only TgAb positive	6 (5%)	1 (1%)	120 (94%)
	Both positive	34 (17%)	2 (1%)	162 (82%)

Abbreviations: TgAb, autoantibodies to thyroglobulin; TPOAb, autoantibodies to thyroid peroxidase; TSH, thyrotropin.

Among the 88 children diagnosed with clinical thyroid disease (11 with hyperthyroidism, 76 with hypothyroidism, and 1 with hyperthyroidism and hypothyroidism), we examined the 73 who were identified as persistently positive for either TPOAb or TgAb prior to diagnosis. Risk of progressing from AITD to clinical thyroid disease was not different between TgAb first and TPOAb first (HR 1.56; 95% CI, 0.80-3.03; *P* = .188). In contrast, children with both autoantibodies appearing at the same visit had an increased risk of diagnosis with clinical thyroid disease (vs TPOAb-first, HR 6.34; 95% CI, 2.72-14.76; *P* < .001). As not all families reported medication with the ICD-10 diagnosis of hypothyroidism or hyperthyroidism, a sensitivity analysis was performed, including only the ones on medication (66/73 children). This did not change the results: the risk of progression from thyroid autoimmunity to treatment for hypothyroidism or hyperthyroidism; TgAb-first vs TPOAb-first: HR 1.49 (95% CI, 0.75-2.99) and both-first vs TPOAb-first: HR 5.74 (95% CI, 2.33-14.13) and for progression to treatment for hypothyroidism only; TgAb-first vs TPOAb-first: HR 1.75 (95% CI, 0.83-3.71), both-first vs TPOAb-first: HR 5.15 (95% CI, 1.88-14.13).

### Autoantibodies to Thyroid Peroxidase and Thyroglobulin Prior to Type 1 Diabetes Diagnosis

TPOAb and TgAb were analyzed in 424 children at the time of type 1 diabetes diagnosis or the nearest visit prior to diagnosis. There were 191 girls with a median follow-up time of 5.1 years (interquartile range [IQR], 2.1-9.3 years), and 233 boys with a median follow-up time of 6.3 years (IQR, 2.4-9.9 years; see Table 2). The cumulative incidence of TPOAb-first was 1.9% compared to 5.9% for TgAb-first at the diagnosis of type 1 diabetes (see Figs. 3, 5, and 6; panels D-F), and the median age at first appearance was 4.7 years (see Table 2).

## Discussion

The incidence and risk factors of TPOAb and TgAb in a cohort of 5066 healthy children from the genetically high-risk TEDDY study revealed increased risk among girls and those with a family history of AITD. The association with HLA genotype varied depending on which of the thyroid autoantibodies was first appearing. A major finding was that seroconversion to a positive thyroid autoantibody started as early as age 10 months for TPOAb first and age 15 months for TgAb first. The consequences of developing TPOAb first vs TgAb first are unclear, as first-appearing autoantibody (among children developing a single autoantibody first) did not alter the risk of either thyroid dysfunction, measured as abnormal TSH or later clinical diagnosis of thyroid disease. The observed upward trend in risk with age confirmed findings from previous prospective studies of children at increased genetic risk or born to parents with type 1 diabetes (18, 19). Although the etiological factors that trigger either TPOAb first or TgAb first remain to be determined, it is a major finding that about half of the children who developed a first autoantibody went on to develop a second by median age 9 years.

Long-term follow-up studies in children born to parents with type 1 diabetes showed that cumulative risk of developing TPOAb by age 8 years was 4.3% (18), and that 10.7% had developed TPOAb by age 20 years (19), while the prevalence of TPOAb by age 10 years among children at increased genetic risk for type 1 diabetes was 4.4% in Sweden and 5% in the United States (20, 21). Our finding of 7.6% of children having either TPOAb or TgAb at the initial screening is in line with these prior reports of prevalence among children at increased risk for type 1 diabetes. Our study, however, adds to the literature by including children both with and without family history of type 1 diabetes, measuring 2 thyroid autoantibodies, and determining the age at first autoantibody appearance with a high level of precision.

The prevalence of thyroid autoimmunity in children after diagnosis of type 1 diabetes has been reported as 14.4% at a median age of 11.3 years or 12.1% at a median age of 12.2 years (22, 23). The lower proportion of children diagnosed with type 1 diabetes and positive for thyroid autoimmunity at or before diabetes diagnosis in our study may therefore be explained by the low median age of 5.9 years at the diagnosis of type 1 diabetes. Seroconversion at a young age is consistent with an increasing occurrence of coexisting autoimmune disorders in children newly diagnosed with type 1 diabetes (24).

Autoimmune endocrine disorders such as AITD, with the exception of type 1 diabetes, are predominately diagnosed in females. Our results, in this cohort of children with genetic risk of type 1 diabetes, demonstrate that the risk for girls was more than twice that of boys, even at an early age. This association between thyroid autoimmunity and sex in our young cohort is notable, as the sex difference for AITD was previously reported to be less apparent in prepubertal children (25).

The increased risk among children with a family history of AITD aligns with previous studies, and increased risk for thyroid autoimmunity has also been shown among children with family members with type 1 diabetes or celiac disease (19, 26, 27). Our finding of an increased risk of thyroid autoimmunity in children who had fathers with AITD is reminiscent of the well-known increased risk for type 1 diabetes in children if the father rather than the mother has type 1 diabetes (28, 29). Furthermore, the HLA DR3/4 genotype is known to confer the highest risk of type 1 diabetes. The increased risk of TgAb-first, but not TPOAb-first, among the HLA-heterozygous individuals raises the possibility that the co-occurrence of type 1 diabetes and clinical thyroid disease may be driven by a TgAb-first subtype, an observation that deserves further exploration.

The predictive value of TPOAb, TgAb, or both, for abnormal TSH and diagnosis of clinical thyroid disease in clinical practice has been a matter of debate. Interestingly, when we examined if the order of appearance of TPOAb vs TgAb was a risk for later disease, no difference was found between the first-appearing autoantibody and later clinical thyroid disease. In contrast, children with both autoantibodies appearing at the same visit had a 6-fold higher risk of developing clinical thyroid disease compared to children with TPOAb-first. Children with only TPOAb and those with only TgAb at the screening visit at age 8 to 13 years did not differ in risk of thyroid dysfunction, that is, abnormal TSH, while the risk was higher for those with both autoantibodies. Therefore, since half of the children with thyroid autoimmunity had both TPOAb and TgAb at the time of the screening visit, a high overall burden of morbidity is expected in this population (30). Our investigation raises the possibility that measurement of both thyroid autoantibodies in children in clinical practice should be considered.

A diagnosis of clinical thyroid disease in infants and young children is rare and is likely to be missed as the diagnosis is more common in older children and adolescents (7). Our finding of thyroid autoimmunity in the very young, although in a selected cohort at risk for type 1 diabetes, stresses the importance of early awareness, to avoid a delay in treatment that may result in an increased risk for neurological damage and growth deficits (31, 32). Further studies of TPOAb and TgAb in children in the general population will be needed to assess the ability of these autoantibody markers to predict thyroid dysfunction and clinical thyroid disease to prevent symptoms.

The HLA-restricted TEDDY population has a higher incidence of autoimmune disease than the general population, allowing us to identify a substantial number of children with thyroid autoimmunity. However, this limits generalization as the cohort is not representative of the general population in each of the respective countries. The TEDDY study is a uniquely valuable resource given its extensive longitudinal follow-up and standardized protocol, but there are constraints inherent to observational studies to consider including unmeasured confounding and sparse data bias (33). Furthermore, retrospective analyses were conducted only in children positive for a thyroid autoantibody at the screening visit, so we were unable to assess if children who were negative at the screening visit were transiently positive at a younger age. Therefore, a major limitation is that additional thyroid autoimmunity may have been missed, as subclinical hypothyroidism can spontaneously remit (34) and the presence of TPOAb, TgAb, or both, can be transient with or without normal TSH levels (35).

Since blood samples were drawn 3 months apart, there were 32 children in whom both autoantibodies appeared at the same visit, therefore we do not know the true order of appearance. It was nonetheless valuable to identify this group of children because they progressed rapidly from 1 to 2 autoantibodies and showed an increased risk for clinical thyroid disease. The low prevalence of Graves disease in children (36) was, at the planning of the study, considered reason for not testing for autoantibodies to the TSH receptor. However, 3 children had low TSH, and 12 had a diagnosis of hyperthyroidism, which suggests that such analyses should be considered in future studies of children at risk.

Taken together, our investigation revealed that thyroid autoimmunity may be triggered in younger children than has hitherto been recognized and that double positivity at seroconversion showed the highest risk for progression to clinical thyroid disease, within an HLA-selected population. It is of interest to further explore the etiological factors that trigger thyroid autoimmunity.

## Data Availability

Data from The Environmental Determinants of Diabetes in the Young (https://doi.org/10.58020/y3jk-x087) reported here will be made available for request at the NIDDK Central Repository (NIDDK-CR) website, Resources for Research (R4R), https://repository.niddk.nih.gov/.

## Committees

^1^Ancillary Studies, ^2^Diet, ^3^Genetics, ^4^Human Subjects/Publicity/Publications, ^5^Immune Markers, ^6^Infectious Agents, ^7^Laboratory Implementation, ^8^Psychosocial, ^9^Quality Assurance, ^10^Steering, ^11^Study Coordinators, ^12^Celiac Disease, ^13^Clinical Implementation.

## Abbreviations

AITD: autoimmune thyroid disease
HLA: human leukocyte antigen
HR: hazard ratio
ICD-10: International Classification of Diseases, Tenth Revision
TEDDY study: The Environmental Determinants of Diabetes in the Young study
TgAb: autoantibodies to thyroglobulin
TPOAb: autoantibodies to thyroid peroxidase
TSH: thyrotropin
